# Spatial transcriptomic brain imaging reveals the effects of immunomodulation therapy on specific regional brain cells in a mouse dementia model

**DOI:** 10.1186/s12864-024-10434-8

**Published:** 2024-05-25

**Authors:** Eun Ji Lee, Minseok Suh, Hongyoon Choi, Yoori Choi, Do Won Hwang, Sungwoo Bae, Dong Soo Lee

**Affiliations:** 1 Department of Nuclear Medicine, Seoul National University College of Medicine, 101 Daehak-ro, Jongno-gu, Seoul, 03080 Republic of Korea; 2 Department of Molecular Medicine and Biopharmaceutical Sciences, Graduate School of Convergence Science and Technology, Seoul National University, Seoul, Republic of Korea; 3 Department of Nuclear Medicine, Seoul National University Hospital, Seoul, Republic of Korea; 4 Institute of Radiation Medicine, Medical Research Center, Seoul National University, 103 Daehak-ro, Jongno-gu, Seoul, 03080 Republic of Korea; 5 Cliniclal Research Institute, Seoul National University Hospital, Seoul, Republic of Korea; 6 Research and Development Center, THERABEST Inc., Seocho-daero 40-gil, Seoul, 06657 Republic of Korea; 7 Medical Science and Engineering, School of Convergence Science and Technology, POSTECH, Pohang, Republic of Korea

**Keywords:** Spatial transcriptomics, Brain imaging, Cell type decomposition, Cell state annotation, Major brain cells, Rare immune cells, Immunomodulatory therapy

## Abstract

Increasing evidence of brain-immune crosstalk raises expectations for the efficacy of novel immunotherapies in Alzheimer’s disease (AD), but the lack of methods to examine brain tissues makes it difficult to evaluate therapeutics. Here, we investigated the changes in spatial transcriptomic signatures and brain cell types using the 10x Genomics Visium platform in immune-modulated AD models after various treatments. To proceed with an analysis suitable for barcode-based spatial transcriptomics, we first organized a workflow for segmentation of neuroanatomical regions, establishment of appropriate gene combinations, and comprehensive review of altered brain cell signatures. Ultimately, we investigated spatial transcriptomic changes following administration of immunomodulators, NK cell supplements and an anti-CD4 antibody, which ameliorated behavior impairment, and designated brain cells and regions showing probable associations with behavior changes. We provided the customized analytic pipeline into an application named STquantool. Thus, we anticipate that our approach can help researchers interpret the real action of drug candidates by simultaneously investigating the dynamics of all transcripts for the development of novel AD therapeutics.

**Supplementary Information:**

The online version contains supplementary material available at 10.1186/s12864-024-10434-8.

## Introduction

Central nervous system (CNS) and central immune system (bone marrow: BM) interactions, specifically brain-immune cross-talk, can occur by a pathway from the skull BM, meninges and their lymphatics, and cerebrospinal fluid (CSF) to the brain parenchyma [1–14] and/or by another pathway from the choroidal plexus (CP) capillary-stroma-epithelium and CSF to brain parenchyma [15–19] in addition to by the classic pathway of crossing the blood‒brain barrier (BBB) [20–23]. In explicit neuroinflammatory diseases such as multiple sclerosis in humans or experimental autoimmune encephalomyelitis (EAE) in animal model, immunoglobulins or immune cells have been considered to enter the brain parenchyma via the BBB [20] of the brain parenchyma or via the brain-CSF barrier of the CP [24, 25], or recently via the arachnoid barrier cell (ABC) layer of skull BM-meningeal lymphatics and CSF/perivascular spaces reaching the brain parenchyma [3–5, 17, 26–28].

Novel immunomodulatory therapy in Alzheimer’s disease (AD) transgenic models, such as 5xFAD mice, should be accompanied by the improvement of cognitive decline associated with aging and/or the amelioration of the transgenes’ adverse effects, such as priming brain cells or immune responses during development and aging. When we inadvertently found the effect of the anti-CD4 antibody while investigating the effect of aducanumab [29] and encountered the probable effect of allogeneic natural killer (NK) cell supplements in AD models [30], we questioned which cells or transcriptomic markers in the brain areas would be the best to predict the outcome of these novel, currently unaccounted therapeutic candidates. In AD mouse models including 5xFAD mice, the surrogate effect markers of previous findings/trials of systemic or intraventricular administration of CD8 + T cells [31], anti-CD8 [32] or anti-CD3 [33] antibodies, Treg cells [34–36] (or for stroke model [37] or DEREG model for traumatic brain injury model [38]), and amyloid-sensitized Th1 cells [39–41] were amyloid plaques/Aβ on immunohistochemistry and transcriptional signatures of major brain cells and brain parenchyma [32, 33, 35] or CP infiltrating cells [25, 42]. As systemically injected cells and immunoglobulins were not examined for their location or biodistribution, direct CNS effects or systemic actions on immune systems were always the alternative to explain the probable effect of novel immunomodulatory therapies, which inevitably led to the insufficient understanding of the target cells and areas. This resulted in inconsistent findings among the reporting investigators.

Single-cell or single-nucleus RNA sequencing (scRNAseq/snRNAseq) based on tissue dissociation and preparation of a single-cell suspension followed by next-generation sequencing allows comprehensive characterization of cell types in the tissue [43–46]. Recently available barcode-based spatial transcriptomics (ST) using the solid-phase capture of RNA on slides, such as Visium® [47–50], HDST [51], slideSeqV2 [52], Seq-Scope [53], or stereo-Seq [54–56], adds a spatial dimension to transcriptomics and enables spatial characterization of genes and cell types through robust regional segmentation of the tissue. Regional and cell-type specific characterization of one or more sections of the mouse brain based on this reliable anatomical segmentation mostly allowed for the comparison of the basal states between groups or even the task-related active states by calcium two-photon imaging and scRNAseq of the visual cortex [57, 58]. Given that ST allows us to obtain genome-wide spatial expression profiles, ST can be considered multiplexed molecular imaging of the brain. We can now use spatial transcriptomic brain imaging to investigate whether a probable immunomodulatory therapy yields its effect on major brain cells (and infiltrating or rare cells of the brain) in each segmented brain area after systemic administration [30]. Biodistribution studies after systemic injection can inform whether immune cells or antibodies enter and directly interact with brain cells; however, if we do not see the immediate presence of cells (usually none) and antibodies (less than 1% of the injected dose), we assumed that they would influence brain cells and pathologic processes. Transcriptomic changes owing to proper novel immunomodulatory therapy will enable us to explore the probable target cells/genes that would have caused or at least be associated with the expected behavioral effects of these therapies [30]. This is especially helpful early in the pursuit of potential new drugs so that investigators can be confident that they are moving in the right direction to modify and optimize new therapeutic candidates. Transcriptomic changes of a region or regions and a cell type or cells in a group are expected to explain the behavioral results of the mouse model. Finally, the transcriptomic changes might predate the behavioral improvements. In both cases, we expect that transcriptomic profiles at a high spatial resolution would excel for drug screening, in sensitivity and target-cell specificity [47, 54], over histological results of immunohistochemistry. Additionally, single-spot ST yields tissue-globally searchable data that can later be reanalyzed repeatedly when the marker gene combination [46, 50, 59–62] becomes available.

To do this, we needed to advance the single-spot RNA sequencing and its analyses using a customized method to derive cell type/state-specific distribution of the Visium sections. Paying attention to the proper dissociation of cell types/states using the optimum/minimum number of genes and cell‒cell interaction (CCI) and cell‒cell communication (CCC) [63], marker gene combinations should be established with the existing public database of scRNAseq/snRNAseq [46, 49, 59, 64–68] and our own data [30]. For both tasks, ready-to-adopt methods are available by the Creative Commons regulations in previous investigations.

Stromal and parenchymal cells of various organs, including the brain, are now known to show common and specific characteristics of cell identity and their ontological characteristics, the best known of which are microglia and perivascular macrophages or resident macrophages [67, 68]. Immune cells of monocyte-derived and resident macrophages have distinctive transcriptomic signatures, which predict their immune roles and tissue integrity-preserving roles per characteristic signatures [69]. This was also the case for T cells, where resident memory T (T_RM_) cells for intestines, effector memory T (T_EM/EMRA_) cells for blood, liver, and BM, and mixed T_RM/EM_ cells for various organs and BM yield their own characteristic transcriptomic signatures that determine their differentiation of T cells in every tissue of interest, dictating their respective functional roles [70, 71]. Unfortunately, neither of these recent cross-tissue, resident immune cell studies [69, 71] included the brain, which mandates our own analysis.

In this investigation, we performed segmentation of brain regions on coronal/sagittal sections per dozens of animals using readily available methods and characterized the common pathologic transcriptomic signatures of 7-month-old 5xFAD mice. Immunomodulatory drugs were administered to these mice to confirm behavioral improvement. ^99m^Tc-hexamethylpropyleneamineoxime (HMPAO)-labeled cell-tracking imaging [72] ruled out the immediate infiltration of NK cells in the brain. Spatial transcriptomic changes in mice were examined after anti-CD4 immunoglobulin administration and expanded NK cell supplement treatment with a dose schedule, which improved Y-maze alternation behavior impairment at this age range in 5xFAD mice. Transcriptomic changes were dissected across areas and cell types/states using publicly available methods and databases, and the analytical pipelines were organized as an application named STquantool. We found that regional/areal gene set-defined type/state-specific cells showed characteristic differences after each trial treatment in a genetic model of AD, 5xFAD mice, upon spatial transcriptomic Visium analysis. Combined brain major cells including neurons, astrocytes, microglia, and oligodendrocytes with their associated types and states and brain resident/infiltrating rare immune cells per region were explored for their distinctive transcriptomic changes among mouse groups to yield their probable association with behavior improvements.

## Results

### Spatial transcriptomic characterization of gene-set-defined type/state-specific major brain cells in 7-month-old wild-type and 5xFAD mice

In total, 35 coronal and 28 sagittal brain sections from 63 mice of either wild-type or 5xFAD background were included in the analysis. A spatial barcode was given for every spot, the unit tissue domain of spatial transcriptomics, and at least 50,000 reads were obtained from each of the 4,992 spots in a capture region. The brain tissues were covered by an average of 3,000 spots across all samples. Using the count matrices computed from Space Ranger as inputs to the reciprocal principal component analysis (RPCA) based integration and clustering pipelines supplied by Seurat (Seurat 4.1; https://satijalab.org/seurat/), the multiple brain tissues were segmented based on their transcriptome patterns [73]. By optimizing parameters such as the resolution of spatial clusters and others, we yielded segmented spatial cluster images in every case, including those with various treatments and manipulations. The treatments and manipulations are listed in Supplementary Fig. 1A and Supplementary Table 1. These included anti-CD4 antibody treatment and NK cell supplement treatment groups. Others were 3-month-old 5xFAD mice, cervical lymphatic ligation, a P301L model with or without amyloid/tau-rich lysate injection, and fingolimod hydrochloride (FTY720) injection with or without lipopolysaccharide (LPS) pretreatment.

The difference between 10 ST data points from wild-type and 11 from 5xFAD animals was compared for each of 14 spatial clusters using 7 to 8 coronal and 3 sagittal sections. For the integration of slides based on RPCA, the transcriptomes of wild-type animals were used as pivots, and the spots from diseased animals were mapped to the PCA space of the wild-type reference [74]. The corrected counts derived from the integration were used to cluster the spots. Spots clusters were visualized in all individual sections and verified for their accuracy in designating the areas according to already-known anatomical correlates (Supplementary Fig. 1B-D). Only the askew sagittal section in a few mice missed the dorsal striatum and instead supplied the septal lobe in a more median position, but the spatial clustering correctly showed the pair of dorsal/ventral striatum in some sections and septal/ventral striatum in others. The spots from each cluster and group were represented by a UMAP plot, a dimensionality reduction method for visualization, and the clusters were well separated in terms of gene expression (Supplementary Fig. 1E, F).

Using the developed platform, STquantool, the representative genes for each cell type were determined by literature or data-driven methods and validated based on spatial gene expression patterns (Materials and Methods). Cell signature scores for each cell type were calculated by the difference between the average expression of the marker genes and that of the randomly selected control genes. Since the cell scores are derived from the curated marker genes and indirectly measure the abundance of the cell type in each spot, it can be postulated that the spatial distribution of the cell scores reflects the spatial distribution of the corresponding cell type. Regional/areal transcriptomes representing major brain cell types/states were then compared between groups by averaging the major cell scores in wild-type and 5xFAD mice using the Wilcoxon rank-sum test (Supplementary Fig. 2). In addition to the major cell types, scores were also calculated in 32 subtypes of neurons [75], several types of astrocytes [76], microglia, and oligodendrocytes [66]. In addition, the reactive state-specific changes and marker genes of astrocytes and microglia were compared between wild-type and 5xFAD animals in 14 brain regions.

To investigate brain regional changes during pathological progression in AD, we obtained ST data from coronal brain sections of the 5xFAD mouse model and age-matched wild-type mice at three and seven months of age. The 5xFAD AD model is known to show amyloid deposition and reactive gliosis from two months of age and synaptic loss and cognitive impairment from four to six months of age (Supplementary Fig. 3A). Amyloid deposition was observed in the adjacent sections of the samples used for ST analysis (Supplementary Fig. 3B). In both coronal and sagittal sections, beta-amyloid levels began to increase at three months of age, and dramatic increases were observed remarkably in the deep cortex, thalamus, hippocampus, and amygdala of seven-month-old 5xFAD mice. The major brain cells were classified into neurons, astrocytes, microglia and oligodendrocytes and their associated cell types (Supplementary Fig. 4A). Neurons were classified according to the reports of Hodge et al. [75] of the Allen Institute with Aeverman et al.’s [59] random forest hierarchical clustering method (NSForest) to define the optimal marker gene combination for neuron subtypes, which was verified by the reports of BICCN [77] and another group’s approach [78–82]. Astrocytes were classified according to the types of white matter-associated and gray matter-associated astrocytes [76] once and again into region-specific astrocytes for the cortex/hippocampus (telencephalon), thalamus, and other brain regions (diencephalon) [49, 83]. Reactive astrocytes and their marker gene combinations were determined by the suggestions of Escartin et al. [84] and other investigators (Habib et al. [85], Ioannou et al. [86], Chamling et al. [87] etc.). Oligodendrocytes and their associated cell types were designated by following an initial report by Marques et al. [66] and were verified by other investigators’ suggestions [86, 87]. Microglia were classified according to their states but not types considering their homeostatic and reactive (microglia with neurodegeneration: MgND [88], disease-associated microglia: DAM [89], lipid-droplet associated microglia: LDAM [90], etc.) states [68, 91, 92], and thus, no subtypes of microglia were assigned. Instead, the aging-related effect on its own or associated with amyloid pathology was examined to show amyloid pathology excluding the confounding effect of aging [93].

**Fig. 1:**
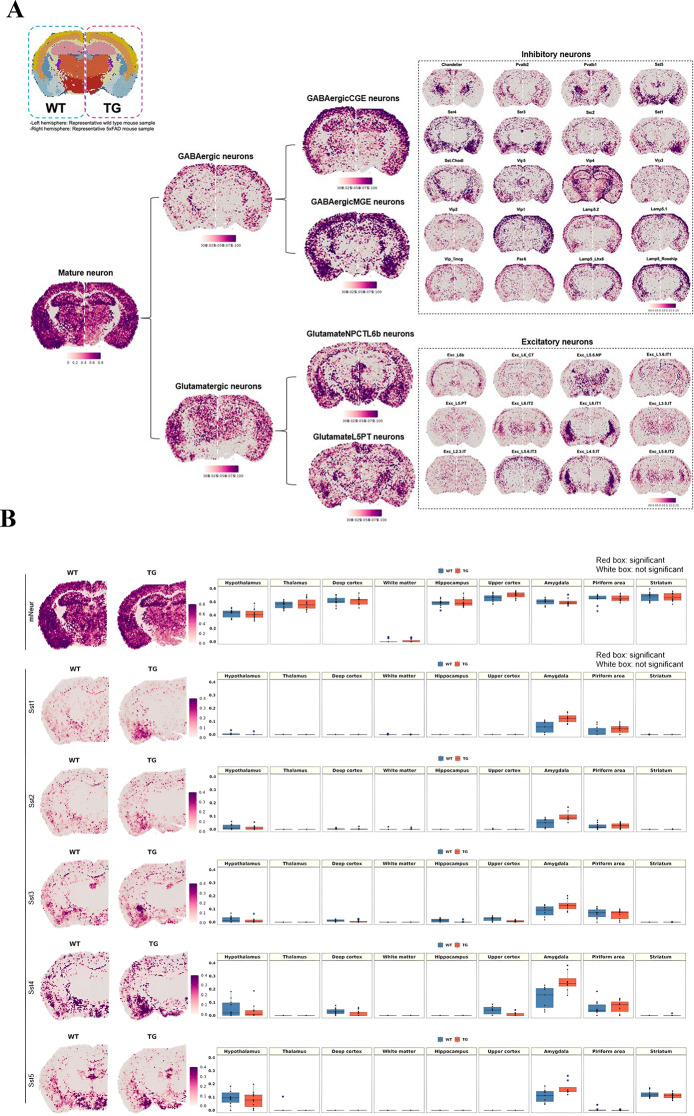
Brain region-specific expression patterns of the signatures of neuronal subclasses and spatial changes in neurons in 5xFAD mice compared to wild-type mice. (**A**) Spatial patterns of diverse neuronal subclass signatures. The left side is a representative slide of a wild-type mouse, and the right side is that of a 5xFAD mouse. Each cell type showed distinct region-specific expression. First, mature neurons were subdivided into GABAergic and glutamatergic neurons, and then the cells were further divided into subclasses to show the regional distribution of subclasses of inhibitory and excitatory neurons. (**B**) Spatial pattern of the neuronal signatures (mature neurons, Sst1, Sst2, Sst3, Sst4, and Sst5; left). Representative images of each group were selected among 10 spatial transcriptome datasets of wild-type mice and 11 of 5xFAD mice. Spatial patterns of the Sst-subclass of inhibitory neuronal signatures of the 5xFAD mice were the most remarkably different compared to those of wild-type mice. The spatial distribution of Sst subclass neurons was similar between wild-type and 5xFAD mice, and the expression of Sst subclasses was higher in 5xFAD exclusively in the amygdala. The boxplot revealed the average module score of Sst-subclass inhibitory neurons, and expression tended to be higher in the amygdala in 5xFAD mice, especially for the Sst4-subclass. Each dot represents a mouse in each group. (mNeur: mature neurons; Sst: somatostatin; WT: wild type; TG: 5xFAD mice; GABAergicCGE: caudal germinal eminence; GABAergicMGE: medial germinal eminence; GlutamateNPCTL6b: near projection, corticothalamic, and layer 6b; GlutamateL5PT: layer 5 and pyramidal tract)

Using the data by Ximerakis et al. [64] for defining cell types, the hierarchical clustering method suggested by Hodge et al. [75] and NSForest (version 2.0) by Aeverman et al. [59], neurons were typed and subtyped into 20 GABAergic neurons and 12 glutamatergic neurons. Their pattern of expression is displayed for wild-type and 5xFAD mice in Fig. 1A. The differences between wild-type and 5xFAD mice in coronal and sagittal sections of 9 areas of interest were quantitatively analyzed (Supplementary Fig. 4B). Subtypes of GABAergic and glutamatergic neurons showed unique patterns in wild-type and 5xFAD mice. The expression patterns of the GABAergic somatostatin (Sst) subtypes in the amygdala differed between wild-type and 5xFAD mice (Fig. 1B and Supplementary Fig. 4C). Increased expression in the amygdala was prominent in the 5xFAD mice compared to the wild-type mice. Notably, the individual genes (*Sst, Nr2f2, Tac1*, and *Moxd1*) tended to be expressed at higher levels in the amygdala of 5xFAD mice among the gene combinations (Supplementary Fig. 5). Furthermore, increased expression of Sst was identified at the protein level in the amygdala and striatum regions but not in the deep cortex, thalamus, or hippocampal regions of seven-month-old 5xFAD mice (Supplementary Fig. 6). The most pronounced increase was found in the amygdala, with only a slight change in the striatum, and the results were consistent with those at the transcript level. Thus, after the accumulation of amyloid plaques in the 5xFAD mice, a marked increase in specific subclasses of inhibitory neuron-associated genes in the amygdala was remarkably identified.

**Fig. 2:**
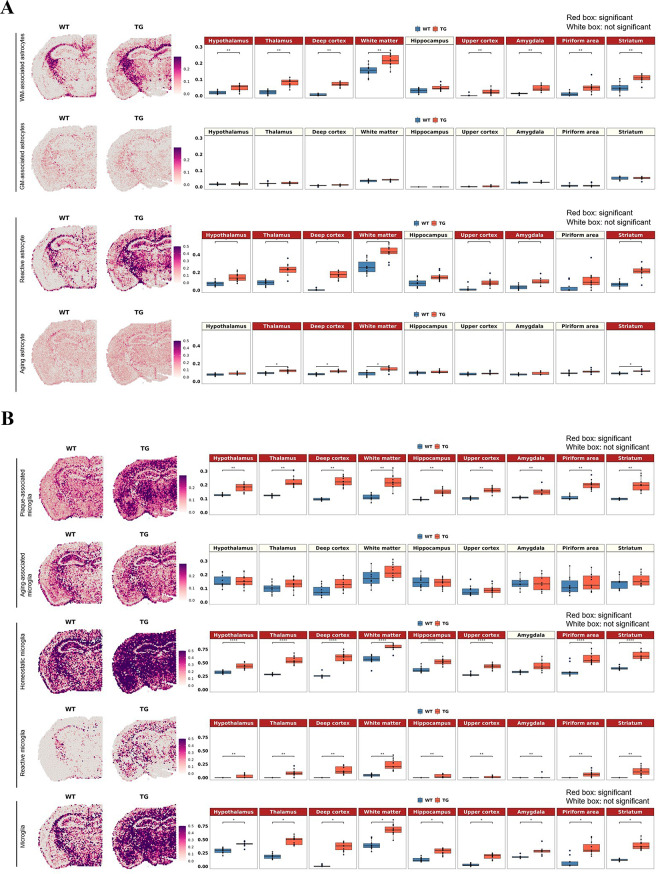
Spatial changes in the distribution of the region- or state-specific signatures of microglia and astrocytes in 5xFAD mice compared to wild-type mice. (**A**) Spatial pattern of the region-specific signatures (white matter-associated and gray matter-associated astrocytes) and the state-specific signatures (reactive astrocytes and aging astrocytes; left). Representative images of each group were selected among 10 spatial transcriptome datasets from wild-type mice and 11 from 5xFAD mice. Boxplot showing average module scores (right). Each dot represents a mouse in each group. The average module score of white matter-associated astrocytes was significantly increased in the white matter and other gray matter regions in 5xFAD mice compared to wild-type mice, but no differences were observed in gray matter-associated astrocyte signatures. Moreover, the average module score of reactive astrocytes showed a similar expression pattern to that of white matter-associated astrocytes, while significant but smaller differences were observed in white matter and several areas in the aging astrocyte signatures. (**B**) Spatial pattern of the state-specific signatures (plaque-associated, aging-associated, homeostatic, reactive, and panmicroglia). The average module score of plaque-associated microglia showed a significant increase in 5xFAD mice compared to wild-type mice, whereas aging-associated microglia showed no difference. Interestingly, both homeostatic and reactive microglia signatures showed a dramatic increase in the average module score in 5xFAD mice. Microglia in general (representing all the state-specific and nonspecific signatures) showed increased expression in all the regions without showing any regional distinctiveness. Bonferroni-adj. *p value < 0.05, **p value < 0.01, ****p value < 0.0001. (WM: white matter; GM: gray matter; WT: wild type; TG: 5xFAD mice)

The difference between wild-type and 5xFAD mice was differential according to the definition (by gene combination to define reactivity) of reactive astrocytes and reactive microglia in their density and distribution (Fig. 2 and Supplementary Fig. 7). Reactive astrocytes and reactive microglia shared gene signatures and were supposed to collaborate to do the job of waste disposal in situ and out of the brain while promoting the interstitial fluid space (ISF) to perivascular/CSF space to meningeal lymphatics. Astrocytes were classified into deep or upper cortical layer-specific and telencephalon- or diencephalon-origin according to Bayraktar et al. [83] and Kleshchevnikov et al. [49]. This classification did not reveal a difference between wild-type and 5xFAD mice (Supplementary Fig. 7A). However, another two types of astrocytes, white matter-associated and gray matter-associated, according to Werkman et al. [76], yielded differences in density and distribution between wild-type and 5xFAD mice. The cell score of white matter-associated astrocytes was significantly increased in the white matter and other gray matter regions in the 5xFAD mice, but no differences were observed in the gray matter-associated astrocyte signatures (Fig. 2A). In addition, reactive astrocytes defined by various ways [84–87, 94] that showed an increase in density in white matter and neighboring gray matter areas (cortex and thalamus) in 5xFAD mice. Their distribution of reactive states was characterized to be diffuse but was prominent around the white matter in coronal and sagittal sections from 7-month-old 5xFAD mice compared with that of wild type mice. Aging astrocytes showed significant but small differences between wild-type and 5xFAD mice in the white matter, deep cortex, thalamus, and striatum (Fig. 2A). Further analysis with individual transcriptomes used as markers for each state-specific astrocyte revealed the following findings. The expression of individual transcriptomes defining white matter-associated and reactive astrocytes showed similar patterns between wild-type and 5xFAD mice, but the dominant individual transcriptomes were different (Supplementary Fig. 8A, B). In the gene combination of white matter-associated astrocytes, *Lyz2, C1qa, Ctss, C1qb*, and *C1qc* were the top five genes with significant differences. In reactive astrocytes, *Gfap, Serpina3n, Vim*, and *C1qb* showed dramatic increases in 5xFAD mice compared to wild-type mice.

Microglia, classified into homeostatic and reactive states [89] and aging-related and plaque-related states [93], showed increased density in wide areas for homeostatic state microglia and reactive microglia (disease-associated microglia, according to Keren-Shaul et al. [89]) and plaque-related (aging-nonrelated but plaque-related) reactive microglia. Interestingly, both homeostatic and reactive microglia showed a dramatic increase throughout brain regions in 5xFAD mice compared to wild-type mice (Fig. 2B). Plaque-associated microglia also showed a significant increase in 5xFAD mice, but aging-associated microglia showed no difference (Fig. 2B). Plaque-associated and reactive microglia shared a similar set of genes (Supplementary Fig. 8C, D). In particular, *Cst7, Spp1, Ccl6*, and *Axl* showed remarkable increases in 5xFAD mice compared to wild-type mice for both microglial signatures. For homeostatic microglial signatures, other genes, such as *Hexb, Cst3, Cx3cr1, Tmem119*, and *P2ry12*, showed dramatic increases in 5xFAD mice. Of note, microglial signatures did not show differences by brain region.

Oligodendrocytes and their lineage cells classified by Marques et al. [66], which comprise mature oligodendrocytes, myelin-forming oligodendrocytes, newly formed oligodendrocytes, committed oligodendrocyte precursors (COP) and oligodendrocyte precursor cells (OPC), showed distinct distribution along the areas, mainly identified in the white matter and faintly in the thalamus and lateral hypothalamus (Supplementary Fig. 7B). A significant difference in newly formed oligodendrocytes in the deep cortex and thalamus was observed between wild-type and 5xFAD mice, but the expression was very low, and the difference was also small. The classification according to Chamling et al. [87], consisting of oligodendrocytes, OPCs and cycling progenitors, also showed similar characteristic distributions. The oligodendrocyte signatures showed relatively little difference between wild-type and 5xFAD mice.

Astrocytes and microglia, specifically white matter-associated astrocytes, reactive astrocytes, plaque-associated microglia, and homeostatic and reactive microglia, tended to increase exclusively in the white matter in 3-month-old 5xFAD mice compared to age-matched wild-type mice. This meant that the changes with the signatures started at an earlier age and occurred around white matter, reflecting a similar result to our previous report [95] (Supplementary Fig. 9). The most interesting finding was that homeostatic microglia also revealed increased expression in most gray matter regions at the later stage of amyloid pathology, similar to the expression pattern of reactive microglia. Increased expression of Tmem119 (a marker for homeostatic microglia) and Cst7 (a marker for reactive microglia) in the gray matter regions, especially in the deep cortex, thalamus, hippocampus, and amygdala, was validated at the protein level in 7-month-old 5xFAD mice compared to 3-month-old 5xFAD mice (Supplementary Fig. 10A). In addition, increased expression of GFAP, S100beta, and Ctss (markers for reactive astrocytes) was confirmed at the protein level in the deep cortex, thalamus, amygdala, and white matter regions (Supplementary Fig. 10B). Thus, the results of verifying the protein expression level were consistent with the ST analysis results.

Finally, DEGs were explored between the groups using the MAST model [96] to find regional differences at the gene level. Of note, we considered gene abundance in addition to the log fold change of mean expression in the spots corresponding to the two groups to classify DEGs in each brain region. Based on the properties of barcode-based spatial transcriptomics, adding abundance information for the corresponding gene within one barcode can increase confidence in identifying DEGs. The spatial expression of individual DEGs in 5xFAD mice compared to wild-type mice was visually assessed by STquantool (Supplementary Fig. 11 and Supplementary Table 2). Venn diagrams of the significantly different transcripts per region were drawn, and the GO terms associated with the genes were visualized as dot plots to examine the differences between wild-type and 5xFAD mice. The reliability of the applied DE analysis was validated by quantitative reverse-transcription PCR (qRT‒PCR analysis) in the thalamus and hippocampus (Supplementary Fig. 12). Among the DEGs from the thalamus, *Hexb, Lyz2, Cst7, H2-K1, Ctss*, and *Gfap* were increased in the thalamus of 5xFAD mice compared to wild-type mice. Additionally, the detected DEGs in the hippocampus, *Scg5, C1qb, Ctss, Hexb, Cst3, S100a6, Cst7, Gfap*, and *Lyz2*, were also significantly increased. Thus, we demonstrated that our transcriptomic approach faithfully captured changes in DE analysis. In 5xFAD mice, both the white matter and gray matter regions showed significant increases in gliogenesis- and glial cell activation-related genes. For downregulated genes-associated pathways, none were detected in the white matter, but ATP biosynthetic process and purine nucleoside triphosphate biosynthetic process were significantly decreased in deep cortex of 5xFAD mice compared to wild type. The DEG-related upregulated and downregulated pathways in other regions are listed in Supplementary Table 3.

### Spatial transcriptomic characterization of rare brain-resident or infiltrating cells in 7-month-old wild-type and 5xFAD mice

Spatial transcriptomic characterization of rare brain cells poses problems of finding the proper unique set of gene combinations for determining these rare cells residing among the confounding major cells. Unlike major cells, the distribution of which is already known, rare cells are low in number and do not have any presumed distribution. Information on the propensity (rarity) of these cells is either derived from scRNAseq studies using dissociated samples from various areas of the brain, even collected from a number of animals, or from the zoomed-in small areas observed by histochemistry. Abundance studies of rare immune cells in the brain reported that the abundance of T cells was 4/mm^3^, that of neurons was 90,000/mm^3^ and that of microglia was 6,500/mm^3^ [97–99]. Other cells such as B cells, monocytes, infiltrating macrophages, dendritic cells (DCs) either conventional or plasmacytoid, or neutrophils were counted and reported for the brain tissue as a whole because all these studies were from scRNAseq analysis using suspended cells from dissociated brain tissue.

**Fig. 3:**
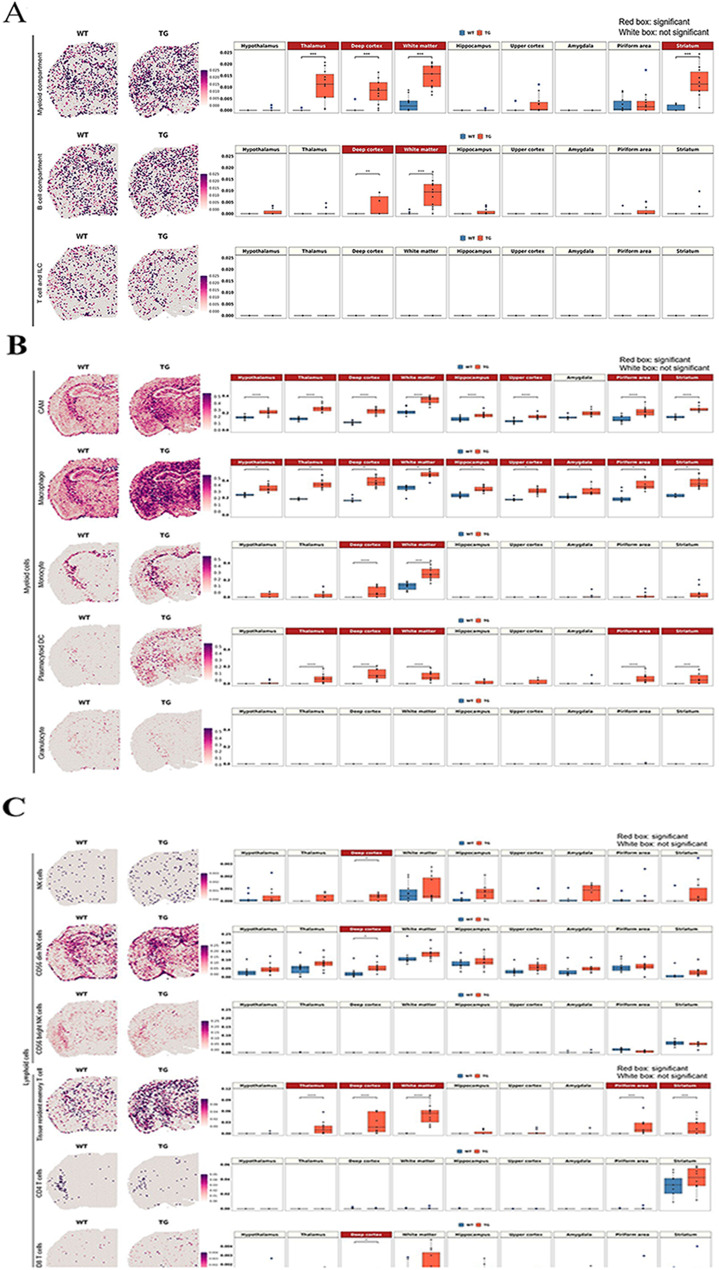
Spatial changes in the distribution of myeloid and lymphoid cell signatures in 5xFAD mice compared to wild-type mice. (**A**) Spatial pattern of the signatures of myeloid, B cell, and T-cell and ILC compartments according to the marker gene combination reported by Dominguez Conde et al. [71] (left) and the boxplot showing the average module scores (right). Each dot represents a mouse in each group. The average module score of the myeloid compartment showed a significant increase in the white matter and gray matter regions adjacent to the white matter, including the thalamus, deep cortex, and striatum. B-cell compartment signatures showed an increase in the deep cortex and white matter. In contrast, T-cell/ILC compartment signatures were low without differences between wild-type and 5xFAD mice. (**B**) Spatial pattern of the subtype signatures of myeloid cells, including CAM, macrophage, monocyte, plasmacytoid DC, and granulocyte according to marker gene combination from Schafflick et al. [68]. Notably, CAM and macrophage signatures showed the most pronounced increase in 5xFAD mice compared to wild-type mice in most of the regions. Monocytes and plasmacytoid DCs were increased in the deep cortex and white matter, and plasmacytoid DCs were further increased in the thalamus, pyriform area and striatum. (**C**) Spatial pattern of the subtype signatures of lymphoid cells, including NK and T cells, according to marker gene combination from Xiemrakis et al. [64]. In the case of the NK cell signature, a significant increase was observed in the deep cortex of 5xFAD mice, which is associated with an increase in CD56dim NK cells. Among T-cell signatures, tissue-resident memory T-cell signatures were higher in 5xFAD mice in the deep cortex, white matter, thalamus, pyriform area and striatum. The CD4 signature was explicit in the striatum in both wild-type and 5xFAD mice, but the expression was too low to show a quantitative difference between wild-type and 5xFAD mice. Bonferroni-adj. *p value < 0.05, ***p value < 0.001, ****p value < 0.0001. (WT: wild type; TG: 5xFAD mice; ILC: innate lymphoid cells; CAM: CNS-associated macrophage; DC: dendritic cells; NK: natural killer)

In contrast to the previous studies that disregarded the heterogeneous distribution of rare immune cells in the brain, solid-phase spot RNA sequencing enabled genome-wide quantification and localization of transcripts, as first documented by Ståhl et al. [47]. In this method and in the now available Visium, a spot has its own count (log1p of the count ratio), which was measured by in situ capture of transcripts in the tissue. However, a spot is composed of a mixture of multiple cells, and it can be difficult to distinguish the transcripts of the rare immune cells from those of the major cell types. In line with this, the problem is to find an appropriate gene (transcriptome) combination to sort out only the specific marker transcriptomes that can discern rare cells from others. Selecting the possible key gene sets defining rare cells with the highest specificity is influenced by the choice of the input data, which are composed of participating cells [50]. For example, T or B lymphoid cells, quite unique with their high propensity for ribosomal protein genes such as *Rpl* or *Rps*, are characterized by any cell-type annotation method to yield candidate marker gene combinations. However, other major brain cells are also equipped with these protein-producing genes expressed in sufficient amounts to appear to be rare brain cells, confounding the presence/density of rare lymphoid cells in any area of the brain. Additionally, since rare immune cells are commonly investigated by combining cell sorting strategies with scRNAseq, the rare cell markers acquired from the subpopulation single-cell dataset may overlap with the major cell type markers. This caused serious overestimation, which was disclosed immediately upon visual assessment. This was also the case despite the use of the recent data available by Schafflick et al. [68] and NSForest by Aeverman et al. [59]. We adopted visual curation to exclude the frankly absurd transcriptomes as marker gene candidates and finally sorted out the rare cells with optimal marker gene combinations to compare wild-type and 5xFAD mice (Fig. 3 and Supplementary Fig. 13).

Immune resident cells were classified in three ways (1) using novel data by Eraslan et al. [69] (Supplementary Fig. 13A) for tissue-specific monocyte-derived macrophages and data by Dominguez Conde et al. [71] (Fig. 3A) for tissue-resident T cells, (2) using the data by Schafflick et al. [68] (Fig. 3B) and markers refined using NSForest 2.0 and (3) using the data by Xiemrakis et al. [64] and refined using NSForest 2.0 by Aeverman et al. [59] (Fig. 3C). The first two reports [69, 71] were derived by using various tissues excluding the brain, while the other two reports [64, 68] were derived by using brain tissues.

Defining marker gene combinations was more intricate for these rare immune resident/infiltrating cells, as they were defined by surface markers in the report of Eraslan et al. [69], in organs/tissues other than the brain, or by transcriptome signatures suited for each study. Although the data were from body tissues, not the brain, in the first approaches, as the tissue stromal cells are included in DEG analysis and assuming stromal cells might be more similar between tissues including brain, transcriptomes of major parenchymal/stromal cells coexpressing with rare immune cells were to be correctly excluded. We chose NSForest to help exclude confounding stromal tissues. Monocyte-derived macrophages include two types, according to Eraslan et al. [69]: one for immune function (MHCII+) and the other (LYVE1+) for vascular integrity and repulsion of infiltrating immune cells. For immune function, specifically for the brain, conventional DCs with MHCII + cells were found to be effective for antigen presentation by adaptive immune cells (T cells and B cells), although border-associated macrophages or microglia were not [100]. We asked whether this surface marker-defined characterization can be translated to mouse (5xFAD) amyloidosis using the signature of MHCII+-related immune-functioning macrophages and LYVE1+-related integrity-charged macrophages described by Eraslan et al. [69] (Supplementary Fig. 13A). Integrity-charged macrophage signature scores were not different among the groups of 7-month-old wild-type and 5xFAD mice in all regions of brain. Spot signatures of immune macrophages were higher in 5xFAD mice than in wild-type mice in the white matter.

Signature gene combinations used in the cross-tissue immune cell analysis by Dominguez Conde et al. [71] revealed no difference between wild type and 5xFAD mice for T cells and innate lymphoid cell T/ILCs. However, significant differences were found among these mice for myeloid compartment cells in the white matter and the gray matter regions adjacent to the white matter, including the thalamus, deep cortex, and striatum and for B cells in the deep cortex and white matter (Fig. 3A).

This result came from the following stepped analysis including the curation procedure. At the first step of curation, individual transcriptomes belonging to the three compartments described by Dominguez Conde et al. [71] were examined visually for their distribution/intensity, and several transcriptomes that were already reported in the literature as signatures for major brain cells and their reactive states were removed, which excluded the background effects of abundant brain cells, eventually yielding marker gene combinations for the three compartments and their cell types. Having removed (1) *Cx3cr1* and *Tyrobp* (microglia) from T/ILCs, (2) *Ighm* (Scheurer et al. [101] for neurons) and *C1qa* (microglia) from the B-cell compartment, and (3) *Trem2* (microglia), *Clu* (astrocytes), *Selenop* (microglia, astrocytes, oligodendrocytes), *Igf1* (reactive microglia and reactive astrocytes), *C1qa* and *Cx3cr1* from the myeloid compartment, the scores of T/ILCs were still not different between wild-type and 5xFAD mice, and the scores of B-cell or myeloid compartments revealed a slight but significant increase in 5xFAD mice compared with wild-type mice. Individual variations within 5xFAD mice could also be recognized upon visual assessment. For individual genes for T/ILCs, localization was prominent for *Cd4* (striatum) and showed little difference regardless of abundance (*Slc4a4, Spry2, Ncam1*, and *Pcdh*9 are abundant), and no difference was observed except for *Pdcd1* (smaller cell fraction in various T cells including Trm/em_CD8 according to Dominguez Conde et al. [71]), which was slightly increased in 5xFAD mice. For the B-cell compartment, the difference between mouse groups, if any, was presumed to be due to *Itgax* and *Fcrls*, both of which were related to aging-associated B cells, and *Fcrls* was related to memory B cells and plasma cells/plasmablasts. For the myeloid compartment, the difference in scores among mouse groups was contributed by *Tyrobp, Lyz2, Fcer1g, C1qc*, and *Apoe*, all of which are related to various types of tissue-specific macrophages and classical/nonclassical monocytes (Supplementary Fig. 14).

The second one by Schafflick et al.’s data [68] was tested for either the marker gene combination recommended by Schafflick et al. according to their supplementary table (log fold change: LFC > 0.5) for 12 border cell leukocytes (including microglia) or the marker gene combination curated by NSForest upon inputting their data. Schafflick’s own data yielded obviously too high intensity for CD4 and CD8 T cells among 12 border-associated leukocytes, such as B1, B2, CD4 T, CD8 T and NK cells, microglia, CNS-associated macrophages (CAM), macrophages, monocytes, myeloid DC (mDC), plasmacytoid DC (pDC) and granulocytes. When we surveyed the constituents of the tentative marker transcriptomes for these inappropriate signatures, ribosomal genes (many isoforms of *Rpl* and *Rps*) were presented as false positive markers of CD4 T and CD8 T cells. This misclassification of marker genes is assumed to be caused originally by the fact that the parenchymal and border leukocytes were included after their selection for CD45 positivity, meaning that they could not exclude the differential expression of these cells from the major brain cells, including stromal cells. Individual transcriptomes per spot were easily observed to disclose whether we chose the highest LFC with adjusted p values for determining marker genes, *Ighm* for b1 cells (also found in the cortex not related to B cells) [101], *H3f3b* (histone protein also nonspecific for the brain) for b1 cells, *Stmn1* for b2 cells (rather brain-wide expression), *Dut* (enzyme for nucleotide and ubiquitous, including brain cells) for B cells and many similar examples (Supplementary Fig. 13B). Although DEG analysis depends upon the input data composition, we tried NSForest on Schafflick’s data and obtained a better marker gene combination. This Schafflick/NSForest analysis yielded improved intensity matching considering the prevalence of cell populations in the brain parenchyma except for b2 cells (still too dense due to *Tuba1b* (tubulin related)) and CD4 T cells (depending heavily upon one transcriptome *Trbc2* (T-cell receptor beta constant 2 but also expressed in the cortex)). The other 10 cell signatures appeared to represent the cell intensity/distribution correctly; however, they also included nonspecific and dense *Apoe* for CAM, dense *Cst3* for macrophages, *Mal* (Myelin And Lymphocyte Protein, implying its localization both in lymphocytes and myelin of neurons) for monocytes, and *Tyrobp* (in association with *Trem2*, a well-known marker for microglia) for both monocytes and pDCs. Upon the application of NSForest, 6 to 10 marker genes were obtained, and zero to three genes were adjusted (kept or removed, meaning curated by operators’ consensus). The application of curated gene combination to our ST samples revealed that CAM and macrophages showed the most pronounced increase in 5xFAD mice compared to wild-type mice globally throughout the brain regions (Fig. 3B). Additionally, pDCs showed increases in the white matter and some gray matter regions. However, the transcriptome density of DCs was considered inappropriate, as it yielded much higher intensity along the entire brain, considering that DCs occupy only 0.14% of myeloid/lymphoid cells of the brain and border, including microglia (0.8% among myeloid/lymphoid cells excluding microglia) [68].

Among lymphoid cell signatures, an increase in tissue-resident memory T cells was inferred in 5xFAD mice compared to wild-type mice (Fig. 3C and Supplementary Fig. 14). However, it is necessary to consider the technical limitations of spot-based transcriptomic analysis for evaluating rare brain cell signatures. It is still unclear which genes specifically define rare cells, while gene combinations may overlap with major brain cells on ST brain imaging.

Using a single gene as a marker would be better and more convenient for designating rare cell types. It was possible to designate infiltrating macrophages derived from circulating monocytes originating from BM (Supplementary Fig. 15). The CD11c surface marker and its gene *Itgax* were used as markers for these cells. Resident T cells were suggested to be CD73 positive, and its gene *Nt5e* was identified by Fang et al. [102]. CD56^bright^ and its gene *Ncam1* are considered to be circulating and immature NK cell markers but are also highly expressed in neurons [103, 104]. Perivascular macrophages cause a great problem in distinguishing them from microglia, and *Lyve1* is the discriminator of pvMϕs and microglia (*Sall1*) [105]. Similarly, for brain major cells, *Trem2* and *Tyrobp* were suggested to be conjoint markers for microglia, and *Cspg* and *Olig2* were expected to represent OPCs, not any other cell types. Homeostatic microglia could have been defined by *Sall1*; however, a transgenic mouse study [105] found that *Hexb* was the better marker for authentic microglia than *Sall1*. The importance of *Aif1* (IBA1) as an activated microglial marker and of *Gfap* as an activated astrocyte marker was disclosed to be nonspecific or at least subtype specific, respectively. Once a marker was well defined for designating rare cell types well discriminated from major brain cells, including microglia and perivascular space (pv) macrophages (and submeningeal macrophages), then that marker in a spot could disclose the fact that the gene signature of that spot might be from the rare cell of interest, but it does not mean that the signature was not from the rare cells if no signal was observed. Genes widely expressed over all cell types but with specific isoforms could be used to define the cell types, and *Prdx* (for peroxiredoxin) was one of the examples (*Prdx6* and *Prdx2* for astrocytes, *Prdx4* for microglia and *Prdx1* for oligodendrocytes) (Supplementary Fig. 16).

To validate the results obtained from the cell signature score based method of measuring cell type abundance, we performed the cell type deconvolution analysis and compared the results between the two methods. The cell type deconvolution method captures the gene expression patterns of cell types from the single-cell reference dataset and predicts the cell type composition in the ST spot, which is a mixture of multiple cells. We performed the analysis mainly for microglia and infiltrating immune cells, which showed significant changes between 5xFAD mice (TG) and wild-type mice (WT) in the signature score-based method. For microglia, the proportions of both homeostatic and reactive microglia were globally increased in the gray and white matter regions of TG mice, which was consistent with the results obtained from the cell signature scores (Supplementary Fig. 17). Minor immune cells, including myeloid cells such as macrophages, monocytes, and dendritic cells, were also upregulated in multiple gray and white matter regions of TG, and the results were similar to those obtained using cell signature scores (Supplementary Fig. 18A, B). However, for lymphoid cell types such as innate lymphoid cells, natural killer cells and T cells, which are rare, the patterns of change between the two methods were inconsistent for a few gray matter regions, while the biological effect may be small due to very low cell abundance (Supplementary Fig. 18C, D). Overall, the results suggest that cell signature scores derived from curated markers are an accurate and reliable measure of cell abundance for the relatively common cell types, while rare cell types require special attention in interpretation.

### Improvement of behaviors with much variation by immunomodulatory therapy of anti-CD4 antibody and NK supplements in the 5xFAD AD mouse model

**Fig. 4:**
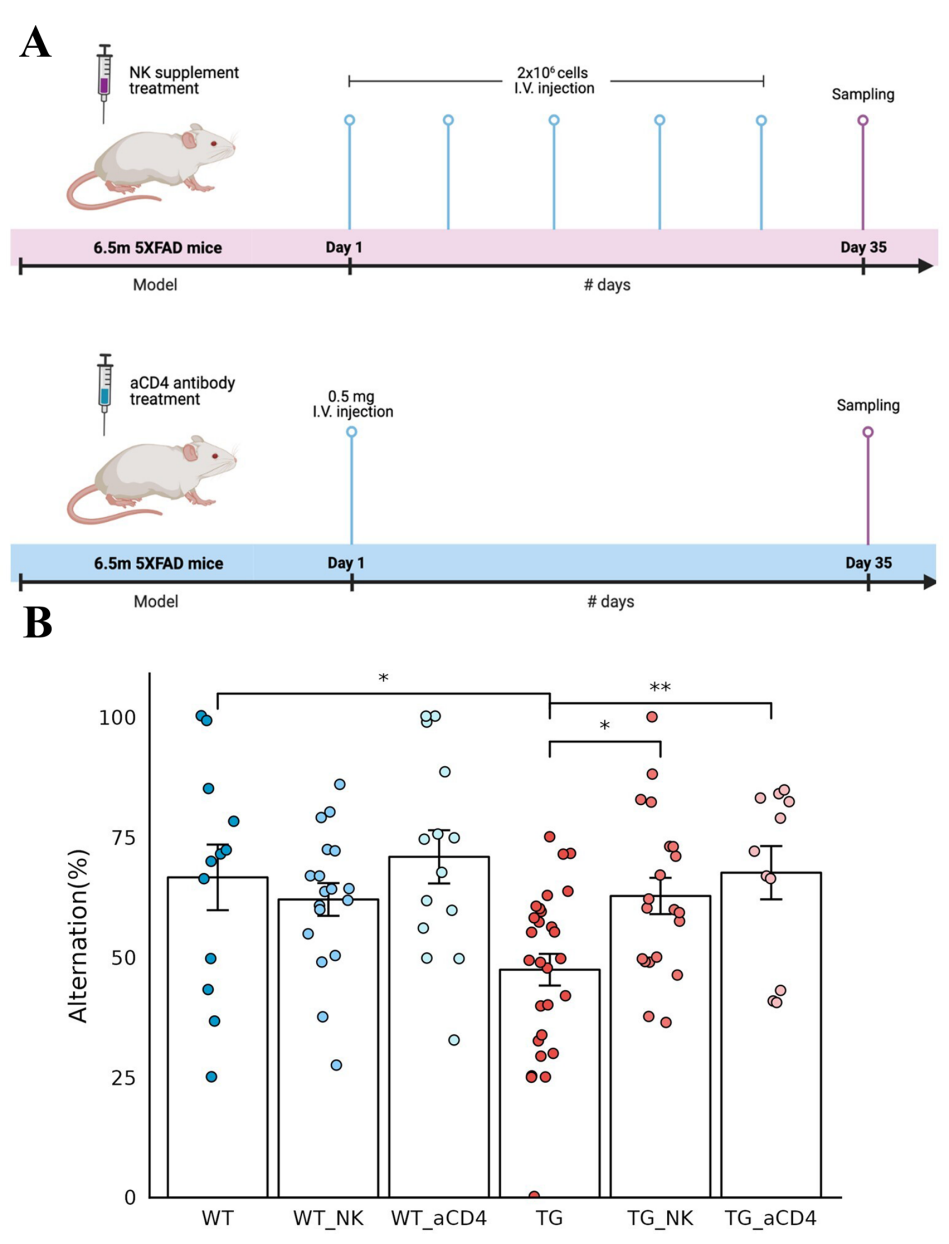
Improved behavior after intravenous administration of NK cell supplement and anti-CD4 antibody in 5xFAD mice. (**A**) Timeline of the experiments for intravenous NK cell supplements (upper) and anti-CD4 antibody (lower) administration in 6.5-month-old wild-type and 5xFAD mice. NK cells (2 × 10^6^ cells/injection) were administered once a week for a total of five times, and anti-CD4 antibody (0.5 mg/injection) was administered once as a single injection. After a month, behavior analysis was performed, and brain tissue samples were obtained for spatial transcriptomic brain imaging analysis. (**B**) The behavioral function of exploring new environments was examined using the Y-maze test and expressed as alternating percentages. Each dot represents a mouse in each group. The alternation rate was decreased in 5xFAD mice compared to wild-type mice, with much variation at this age in wild-type and 5xFAD mice. The alternation percentage score of 5xFAD mice increased significantly after injection of NK cell supplements and anti-CD4 antibody treatments compared with that of 5xFAD mice without treatments. Wild-type mice also showed variation; however, their alternation scores were not different between the no treatment and either treatment group. Wilcoxon *p value < 0.05, **p value < 0.01. (aCD4: anti-CD4 antibody; WT: wild type; TG: 5xFAD mice; WT_NK: NK cell-treated wild type; WT_aCD4: anti-CD4 antibody-treated wild type; TG_NK: NK cell-treated 5xFAD; TG_aCD4: anti-CD4 antibody-treated 5xFAD)

During a preliminary behavioral study to prove the effect of aducanumab, pretreatment with anti-CD4 antibody caused a larger degree of changes in alternation scores in the control animals (meaning higher improvement in the group of animals treated with anti-CD4 antibody) [29, 30]. Three batches of several animals with anti-CD4 antibody treatment reproduced the previous groupwise behavioral improvement with similar variation (67.7% ± 18.4%) at 7 months of age in 5xFAD model mice (Fig. 4). We assumed that anti-CD4 antibody treatment modulated the systemic adaptive immune system, as transgenic insertion of five types of mutated human APP/PS1 genes would have caused immune disturbance due to their presence in the mouse chromosome. The presence of human mutated genes would have resulted in brain-immune interaction dysfunction as well as plaque-prone amyloid burden in animals. Spatial transcriptomic analysis was considered to reveal the eventual response of brain cells, either major or rare resident and infiltrating immune cells, if any.

In another preliminary study with APP/PS1 model mice using the water maze with expanded NK cell supplements derived from the spleen of wild-type BALB/c mice, anecdotal cases of behavioral improvement were observed (data not shown). Three batches of allogeneic NK cell supplements, as 5xFAD mice are on a B6 background, reproduced the behavioral improvement of alternation scores on Y-maze tests on average, however, with much variation (Fig. 4). Much variation in both the anti-CD4 antibody treatment study and NK supplementation study indicated that 5xFAD mice at 7 months of age were undergoing their own course of aggravation of the pathological changes of Aβ oligomer insults and amyloid plaque burden, resulting in later pathological and behavioral dysfunction at approximately 12 months of age or later. Spatial transcriptomic analysis was also considered to reveal the regional and cell-type specific changes of transcriptomes of major and rare brain cells corresponding to each individual mouse’s degree of behavioral dysfunction in the NK supplement-treated group.

### Regional cell-type/state-specific transcriptome changes in 5xFAD mice compared with wild-type mice after intravenous administration of NK cell supplements

Three mice with higher alternation scores on the Y-maze test were selected for both the saline-treated and NK cell supplement-treated groups (Supplementary Fig. 19). Coronal/sagittal brain sections of these mice were subjected to Visium analysis. Each group was paired to the same plates so that the batch effect of the read per slide would be minimized. Using 30,000 to 50,000 counts per mouse, we retrieved the count data, which were normalized for their total count, and log1p of the ratio data were used for further analysis. Spatial clustering allowed anatomical segmentation to yield 14 regions with almost similar sizes (Supplementary Table 4). Cell type- and state-specific marker gene combinations were also used to analyze cell-specific and/or cell state-specific changes after NK cell supplement treatment. For the 5xFAD case with NK cell supplements, one mouse with a very high behavior score was chosen as the ‘behaviorally best’ representative of the group, and another mouse with a very low score was chosen as the ‘behaviorally worst already at 7 months of age’ representative. This essentially allowed us to examine the transcriptomic changes according to the behavioral impairment of the 5xFAD mice at the age of 7 months. NK cell supplements contributed at least to the widening of the distribution of scores of behavioral impairments at this middle age.

**Fig. 5:**
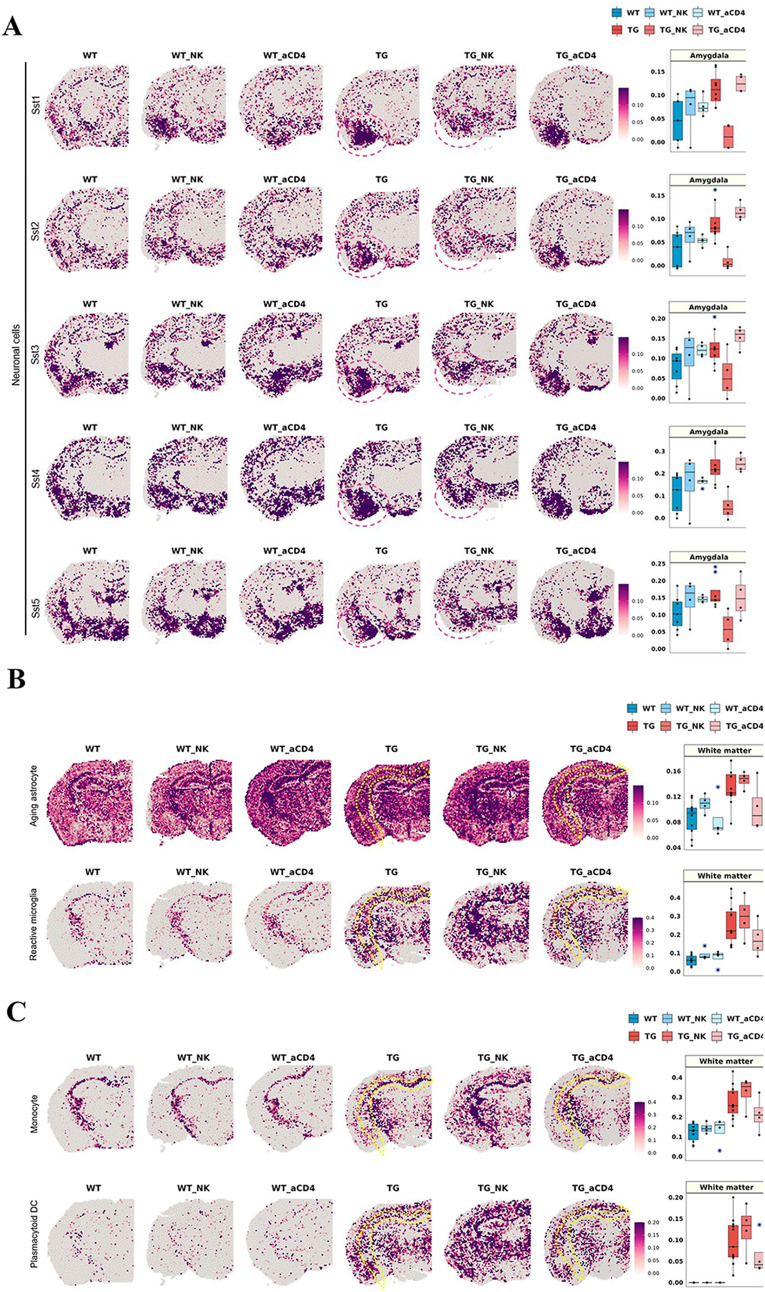
Brain region-specific transcriptome changes in cell signatures after NK cell supplementation and anti-CD4 antibody treatment in 5xFAD mice. (**A**) Spatial pattern of the signatures of somatostatin (Sst)-inhibitory neuronal signatures (Sst1, Sst2, Sst3, Sst4, and Sst5; left) and the boxplot showing the average module scores in the amygdala (right). Each dot represents a mouse in each group. The average module scores of Sst neuronal subclasses tended to decrease specifically in the amygdala after administration of NK cell supplement in 5xFAD mice. Interestingly, the Sst neuronal signatures, which were increased in expression in the amygdala of 5xFAD mice, were decreased to the expression level in wild-type mice by NK cell supplements. In contrast, the module score was not different between the no treatment and anti-CD4 antibody treatment groups, while there were differences between the no treatment and NK cell supplement groups. (**B**) Spatial pattern of the signatures of state-specific glial cells (aging astrocytes and reactive microglia; left) and (**C**) immune cells (monocytes and plasmacytoid DCs) and the boxplot showing the average module scores in the white matter (right). The expression of state-specific subtypes of glial cell and immune cell signatures, which showed a significant increase in 5xFAD mice compared to wild-type mice, tended to slightly decrease in the white matter after anti-CD4 antibody treatment. Considering that NK cell supplementation showed no appreciable differences in glial cell and immune cell signatures, anti-CD4 treatment effects on these cell subtypes of state-specifics looked real. Expression, however, did not decrease to the level observed in wild-type mice. In summary, NK cell supplementation and anti-CD4 antibody treatment affected different state/type-cell signatures and brain regions, respectively. (aCD4: anti-CD4 antibody; WT: wild type; TG: 5xFAD mice; WT_NK: NK cell-treated wild type; WT_aCD4: anti-CD4 antibody-treated wild type; TG_NK: NK cell-treated 5xFAD; TG_aCD4: anti-CD4 antibody-treated 5xFAD; Sst: somatostatin; DC: dendritic cells; NK: natural killer)

GABAergic Sst subtype neurons showed a significant decrease after NK supplementation in the amygdala, which showed an abnormally increased signature in 5xFAD (*n* = 11) compared with wild-type mice (*n* = 10) (Fig. 5A). Among the Sst neuronal signatures, *Sst, Tac1*, and *Nr2f2* showed dramatic decreases in the amygdala after NK cell supplement administration in 5xFAD mice, but there were no distinct differences in the other regions (Supplementary Fig. 20A, B). The Sst-expressing neurons in the cortex are known to contribute to modulating cortical circuits, synaptic plasticity and maintaining spatial working memory [106]. Patients with AD exhibited low Sst expression in the cortex and hippocampus. However, the function of Sst-inhibitory neurons in the amygdala remains poorly understood [107]. No significant difference was found either in the mature neuron score or in any other subtype of neurons other than Sst neurons between 5xFAD mice without NK supplements and those with NK supplement treatment. Thus, normalization of excitatory and inhibitory neuronal imbalances in the amygdala may improve behavior function. Further investigation of the neurons in the amygdala region could play an important role in understanding the pathology of AD and in providing therapeutic directions. Additionally, the NK cell signature tended to increase after administration of NK cell supplements exclusively in the white matter region of 5xFAD mouse brains (Supplementary Fig. 21A). The change in the module score level was not observed with the anti-CD4 antibody treatment, which was in contrast with the change after NK cell supplement treatment (Supplementary Fig. 21B). The signatures of astrocytes, microglia, and oligodendrocytes did not show any difference. Additionally, no difference in rare brain cells, either resident or infiltrating, was observed (Supplementary Fig. 22). The biodistribution of ^99m^Tc-HMPAO-labeled NK cells was examined using SPECT/CT to determine how systemically injected NK cells caused changes in the brain (Supplementary Fig. 23). Within 1 h after injection, the labeled NK cells were mainly taken up by the liver, and this radioactivity decreased gradually by 16 h. Of note, no definite brain uptake of the labeled NK cells was observed with the resolution of SPECT/CT images. Thus, NK cells may have caused changes in brain cells at the transcriptional level indirectly via cytokines or other secretory factors released by NK cells and/or inherent peripheral immune cells influenced by supplemented NK cells.

### Regional cell-type/state-specific transcriptome changes in 5xFAD model mice compared with wild-type mice after intravenous anti-CD4 antibody treatment

Three mice in the anti-CD4 antibody treatment group were selected, and their coronal brain sections were plated on Visium slides. The frame of sample distribution on the quadrants of each Visium slide was the same as above for NK cell supplement treatment. Further analysis of transcriptomes per spot and spatial clustering and designation of transcriptomes to the approximately 3,000 spots were also the same.

Among major brain cells, state-specific glial cells, such as aging astrocytes and reactive microglia, which showed a significant increase in 5xFAD mice compared to wild-type mice, showed a slight decrease exclusively in the white matter after administration of anti-CD4 antibody in 5xFAD mice (Fig. 5B). In the gene combination of aging astrocytes, the expression of *Lgmn, Gsn, Mt1, Fcrls*, and *Hexb* was noticeably decreased in the white matter after anti-CD4 antibody administration in 5xFAD mice, and expression decreased slightly in the other regions (Supplementary Fig. 20C, D). In the reactive microglial signature, *Cst7, Spp1*, and *Cd9* showed a decreased pattern throughout the region, while other genes, such as *Axl*, *Csf1*, and *Ccl6* showed decreased patterns mainly in the white matter (Supplementary Fig. 20C, D). Interestingly, the CD4 T-cell signature tended to decrease slightly in the deep and upper cortex (striatum) after anti-CD4 antibody treatment (Supplementary Fig. 21B). However, mature neuronal signatures showed typical and very similar patterns of distribution between 9 major brain regions and within each region irrespective of whether the sample was from wild type, 5xFAD, wild type with anti-CD4 antibody treatment or 5xFAD with anti-CD4 antibody treatment mice (Supplementary Fig. 22A). No significant differences in other types of glial cells, including white matter-associated astrocytes, reactive astrocytes, plaque-associated microglia, homeostatic microglia, and oligodendrocytes, were found, meaning that all 10 wild-type mice and all 11 5xFAD mice could not be differentiated between no treatment and anti-CD4 antibody treatment (Supplementary Fig. 22B, C). The difference between wild-type and 5xFAD mice was sustained but did not reveal any dramatic effect of anti-CD4 antibody treatment.

Rare brain cells, resident or infiltrating, were distinguished between the no treatment group and the anti-CD4 antibody treatment group. The expression levels of monocytes and pDCs, which showed a significant increase in 5xFAD mice compared to wild-type mice, tended to slightly decrease only in the white matter after anti-CD4 antibody treatment (Fig. 5C). In both monocyte and pDC signatures, the expression of *Tyrobp* was dramatically reduced throughout the brain, whereas *S100a4, S100a10*, and *Mal* in the monocyte signature showed decreased expression patterns exclusively in the white matter after anti-CD4 antibody treatment. A reduction in the expression of individual genes by the anti-CD4 antibody was mainly identified in the white matter region (Supplementary Fig. 20C, D).

While NK cell supplementation showed no appreciable differences in immune cell signatures, the fact that anti-CD4 antibody treatment showed effects on subtypes of immune cells is noteworthy. However, the expression level was not decreased to that observed in wild-type mice.

### Methods to scrutinize spatial cell-type and cell-state specific changes upon the platform of setting norms and characterization of abnormality of a test mouse

As spatial distribution is critical for characterization of a new mouse specimen for their status of normalcy, pathology, and response to therapy, the specimen can be on every section, but on a limited number of coronal sections (per monkey samples in a report by Chen et al. [55]) or sagittal sections. To acquire representative information regarding mouse groups, we combined multi-individual mouse sections to yield the apparently correct spatial segmentation. Each region was then prepared to present their norms for various scores for cell types, cell states and response to the tentative immunomodulatory drugs. We tried to establish methods to reveal the regional cell-type/state-specific norms and their probable changes by drug intervention. We set up norms for normal mice using wild type mouse data genotype, and then the effect of the age or the influence of drug treatments were characterized. For example, the presence of anomaly were examined for individual mice according to their disease states (5xFAD mice of certain age with diverse behavior/Aβ abnormality, P301L mice with no behavioral/pathological abnormality) and the effect of therapy (anti-CD4 antibody or NK cell treatments).

Cell types should have been annotated to the then-best knowledge of the scientific community based on the resource reports in the literature up to the date of this investigation run by trial-and-evaluation and then the choice; for example, for neurons and neuronal subtypes, Hodge et al.’s report [75] was adopted as is or after NS Forest [59] to define 20 GABAergic neurons and 12 glutamatergic neurons. Available data were downloaded from specific sites or supplementary tables of each report. Thus, for example, Scng-VIP neuron subtypes described in a more recent report by Bugeon et al. [57] were ignored but later can be reanalyzed with the current Visium data by specifying their markers along the regions segmented after integration of slides using RPCA. For astrocytes and microglia, the reactive state signature was surveyed by scrutinizing the counts of transcriptomics at each spot according to the reports by Keren-Shaul et al. [89], Friedman et al. [91], Grubman et al. [93] and others. Coexpression of the same transcriptome by astrocytes and microglia, such as *Apoe, Gfap, Tspo* and others, was removed from the tentative marker gene combination. The same procedure was performed for astrocytes and oligodendrocytes or microglia and oligodendrocytes. Oligodendrocytes and their lineage cells did not have a ‘reactive’ transcriptome signature. Signature transcriptomes between reactive and homeostatic microglia (and astrocytes) were also surveyed for their conjoint expression between both states. Homeostatic transcriptomes were designated to exclude the signature of reactive transcriptomes and vice versa, referring to literature reports by Prinz et al. [108–114], and Kim et al. [105], so that *Sall1* and *Hexb* were used to measure the abundance of microglia as each microglia express these genes constitutively while assuming that these transcriptomes did not increase in quantity per microglia when reactive [105, 113]. As spatial transcriptomic imaging using Visium yielded a linear (semi)quantitative (due to log1p transformation for further processing using Seurat 4.1)) metric, no fractional presentation was adopted to disclose that homeostatic microglia were increased in quantity of signature per spot in 5xFAD mice compared with wild-type or P301L mice.

Quantification was performed for 9 regions (hypothalamus, thalamus, deep cortex, white matter, upper cortex, hippocampus, amygdala, piriform area, and striatum) for major cell types, including neuron subtypes, reactive and homeostatic glial cells, astrocyte subtypes, oligodendrocyte lineage cell subtypes and rare resident/infiltrating immune cells. For regions, for example, white matter-associated astrocytes or white matter-localized microglia were quantified and correlated with white matter-localized oligodendrocyte lineage subtypes. Dense and thus intense quantities of mature oligodendrocytes could be compared among mouse groups. In contrast, diffuse and sparsely distributed rare immune cells were found in three compartments by Dominguez Conde et al. [71], four types by Xiemerakis/NSForest [59, 64], and 12 types by Schafflick et al. [68]. Tissue resident macrophages by Dogra et al. [115] and Eraslan et al. [69] were also quantified for intensity to yield the difference for each region among mouse groups. The Wilcoxon rank-sum test was used to determine the significance between pairs of groups (uncorrected p value or corrected by three for paired group comparisons). Beyond group comparison, an anomaly detection procedure (or confirmation of normalcy meaning no difference found on any regional, cell-type, cell-state specific signature by density per region) was performed for each mouse.

Once a region and cell type and its state were found, we performed DEG analysis to find the transcriptomes of interest in each brain region. Then, the association of discovered genes with biological pathways was examined using an overrepresentation test based on evidence-driven databases. This was to determine the significance of the found transcriptome designating their functional role in pathology (amyloid pathology or tauopathy, glial cell dysfunction, etc.), physiology (aging) and their participation in the response to tentative immunomodulatory therapy. Considering the diversity of behavior improvement after anti-CD4 antibody and NK cell supplement treatments and the unpaired nature of the Visium study, we could only detect the treatment effect (and non-effect) of the transcriptome signature of regional cell types/states upon treatment per individual. Transcriptomes of the marker gene combination that we used were all checked for their individual transcriptomes to elucidate key transcriptomes for specifying type/state characteristics or therapy effects. We also tried to assess their individual contribution to this specification to find one, two or more distinctive transcriptomes to predict their presence in each spot. This means that curation by operator in addition to the readymade Wilcoxon, logistic, or NSForest methods was used in at least two steps, first to choose a seemingly optimal combination ruling out cross-expressing, background, or confounding genes and then at last to find the succinct combination of transcriptomes for cell-type/state annotation or if any, the sole transcriptome (Supplementary Fig. 2). The above pipelines for dissecting cell-type- and cell-state-specific regional transcriptomic changes can be readily implemented with our in-house application STquantool, which facilitates the visualization of spatial gene expression and enables quantification across multiple transcriptomic datasets.

## Discussion

In this investigation, we used ST for its superiority over scRNAseq/snRNAseq to localize the specific transcriptomic signature of cell type or cell state in almost 5 thousand spots, among which 3,000 or more spots harbored either coronal or sagittal sections of brain tissue. Before going further to use this transcriptomic signature to disclose the effects of novel but unproven neuromodulatory treatments, we trimmed the method of the use of this Visium-based ST imaging to elucidate regional, cell-type/state-specific changes. The method for choosing one or two optimal transcriptomic marker combinations among so many possible combinations was adjusted to yield the best contrast between cell types/subtypes using the literature resources and our in-house method of curation. A simple and easy method to sort out the candidate transcriptomes was set up to ensure that we found the best cell type/state annotation methods for either abundant brain cells or rare immune cells. The challenge was to separate 4 or more major brain cell types and their subtypes with transcriptome combinations and to define rare immune cells for their exact propensity and distribution/location. Stahl et al.’s [47] suggestion of counting the transcriptomes per spot using the original ST Visium methods and Tirosh et al.’s [44] approach to generate cell signature scores based on the curated marker genes and comparing them between mouse groups with genuine or sham treatments worked well for this endeavor. We overcame the problem of high dependence of this endeavor on the choice of tentative marker gene combinations varying upon the diverse input data derived from the preliminary DEG studies using single-cell data of brain tissues [67, 68, 108] and even tissues other than brain tissues. Assessing the sophisticated use of the public database and scrutinizing the individual transcriptomes visually by the operators (neuroimaging experts) were essential. Curation by operators is heuristic at best and is surely subject to operator arbitrariness; however, this was eventually the key step to enhance the authenticity of the observation of large number of cells (2 to 10 per spot) admixed in spots and a dozen specimens from individually different but syngeneic mice. From the neuroimaging perspective, integrated single spot imaging (100 µ x 100 µ x 10 µ) containing an average of 5 cells (2 to 10) in each unit domain did not have a significant batch/individual variation effect to confound further analysis, as we observed dozens/hundreds of spots at the same time, and the batch effect was corrected during sample integration. With this visual investigation, we soon became confident that spatial transcriptomic brain spot imaging with visual assessment and its quantitative analysis using the framework of voxel (spot) imaging of mouse/human brains was suitable for the evaluation of the effect of certain drugs/treatments for disease-course modification in dementia mouse models.

The transcriptomic signature of brain cells could clearly segment every section from the mice, regardless of disease or treatment status, taking advantage of 22,000 or more transcriptomes per cell to identify the cell type/state with thousands of variable transcriptomes. Unlike functional neuroimaging such as functional magnetic resonance imaging (fMRI) or positron emission tomography (PET), which needs coregistration and segmentation considering individual variation for further analysis, the segmentation of neuroanatomical entities on Visium could be performed without any more assumptions, except that functional regional entities could be determined by transcriptomes belonging to spots and their conglomerated spots make explicit functional regions. An eccentric case of regionally remote but similar transcriptome composition was observed in that the cortical amygdala and subcortical septal lobe were categorized as the same cluster on sagittal section, but excluding this exception, all the other spatial clusters were within the expected anatomical border definition (https://connectivity.brain-map.org/3d-viewer?v=1&types=IMAGEPLANE&IMAGEPLANE=imageplanes). Thus, spatial or regional representation of characteristic changes related to pathology and treatment response could then be described and quantified. Finding marker gene combinations to define the spots as belonging to certain functional regions of interest then could be achieved by finding the optimal or best combination, which would be appropriate and succinct.

Determination of the best annotation of neurons and other major brain cells was initially dependent upon previous reports mainly derived from nonspatial scRNAseq/snRNAseq analysis [63, 73, 80, 116]. In these previous studies, the spatially expected designation of cells was suggested as a success of cell clustering, raising concerns that there was no gold standard information regarding their true location; nevertheless, the cell clustering and annotation allows assignment of regional identity of brain cells based on anatomical region-specific marker genes. ST brain imaging obviated this concern. In ST imaging, however, there remain two major problematic ambiguities for spatial clustering and cell type/state identification per spot. The first one is spatially agnostic annotation by transcriptome signature, which previous researchers tried to solve by sampling regions of brain such as posterior isocortex, hippocampus (or hippocampal formation), striatum, thalamus and hypothalamus, etc., in the reports of Saunders et al. [65] or Chai et al. [117]. This problem was easily solved by ST imaging using Visium of 3 to 5 thousand spots, which allows capturing transcriptomic changes across the broad area of the brain. Now, imaging with a resolution of 100 µ x 100 µ on 2D is available, allowing easy segmentation; this differs from fMRI/PET in that the huge multiplexing capability of ST brain imaging allows almost infinite reanalyses using combinatorics. The second is cell/state identification per spot by using the transcriptomic signature of the marker gene combination determined by previous DEG studies using detached and sometimes surface-marker-sorted brain cells. When scRNAseq/snRNAseq was used for detached brain cells to determine the effect of drug/treatment on those brain cells, lack of spatial localization was the major hurdle blocking the understanding of the role of any treatment. In situ hybridization of immunohistochemistry complemented transcriptomic global/regional brain signatures to address this, but without reassuring results to explain the therapy effect. ST brain imaging solved this problem. As shown in this study, ST brain imaging is equipped with the expression profile per spot for the entirety of genes of the individual cells localized on each spot, and the data could be analyzed in an unsupervised fashion without any assumption or in appropriate cases by using a priori knowledge derived from the literature resources of scRNAseq/snRNAseq. Considering the challenges and difficulties in overcoming these problems, we streamlined the use of visual reading by expert operators called curation. The steps required for curation were kept minimal and practical, and it was performed initially to exclude nonspecific and cross-expressing transcriptomes between major cells, and finally to exclude cross-tissue, stromal cell-dependent and confounding background signatures. It would have been better to base curation on individual transcriptomic features of any types of cells for their association with disease states or drug/treatment responsiveness.

To tackle these problems, we asked how we could use individual mouse ST brain imaging data to determine the disease states, which are variable even in syngeneic animals, and the variable treatment responses affecting major and rare brain cells. Taking advantage of the automatic segmentation results for groups of individual mouse specimens, irrespective of section planes and stereotaxic coordinates, we tried to individualize the transcriptomic features of each individual specimen compared with the norms we constructed. Comparing regional, cell type/state-specific transcriptomic signatures using visual and quantitative decisions of an individual mouse with norms was performed. This analysis method allowed for the individuation-based evaluation of animals for their behavior correlates. We were able to obtain and reproduce a wide variety of behavior metrics, which is in this study included the alternation score on the Y-maze; the Y-maze alternation scores of 7-month-old wild-type mice ranged widely as well as those of 5xFAD mice, but those of 8.5-month-old wild-type mice converged with smaller variation to lower values, meaning commonly poorer performance at this age even in wild-type mice. After anti-CD4 antibody treatment, the variation was sustained with a slight improvement in their average scores (Fig. 4B). After NK cell supplement treatment, variation was also sustained, with slight improvement in their average scores (Fig. 4B). We assumed that these behavior variabilities are the keystone for proving the feasibility of tentative novel immunomodulatory treatments and that we would find that the mouse behavior scores concord with the transcriptomic signature [57]. NK cell-treated 5xFAD mice with higher Y-maze alternation scores definitely showed that their amygdala GABAergic Sst neuronal subtypes decreased in intensity (Fig. 5A). This decrease (or increase, if any) did not prove the efficacy of NK cell supplement treatment on 5xFAD mice but definitely disclosed that transcriptomics of the neuronal subtype of that region were correlated with the degree of behavior impairment. More importantly, this meant that many other neuronal subtypes, other homeostatic or reactive glial cells and their subtypes, did not show any change in intensity over all the regions examined on these sections despite the improved behavior score. Anti-CD4 antibody treatment recapitulated only a slight decrease in specific immune cell signatures in the white matter, but beyond this finding, no other discovery of drug-responsive transcriptomic changes in any region or in any cell type or cell state was found. This was even observed on individual interpretations both visually and quantitatively for each mouse (Fig. 5B and Supplementary Fig. 21). We could say that anti-CD4 immunoglobulins did not affect the transcriptomic signatures of major brain cells (on this single coronal section), and this was also the case with rare immune cells. Due to the lack of Y-maze score measures of the anti-CD4 antibody-treated wild-type and 5xFAD mice, behavior correlation could not be reported here.

The interpretation of rare immune cell signatures for the localization of immune cells presented different challenges from major brain cells. First, due to the intrinsic limitations of Visium, rare cell transcripts may not be well captured compared to the abundant cell type. Additionally, Visium captures a mixture of transcripts from multiple cells and lacks single-cell resolution. Therefore, it relies on cell type abundance estimation tools, which may be less reliable than image-based ST methods that capture transcript expression at the single-cell level. Nevertheless, we attempted to overcome the limitations with several strategies. The first was to remove the background effects of major brain cells. Homeostatic and reactive microglia and their coexpressed transcriptomes between microglia and infiltrating monocytes were the major challenge but were easy to remove, and astrocytes and oligodendrocytes followed by reactive glial cells expressed the same/similar transcriptomes. Double-checking the unique transcriptomes and their combinations was attempted with the data by Ximerakis et al. [64] and Schafflick et al. [68]. based on brain tissue studies. The study by Schafflick et al. [68]. used cells sorted by FACS for CD45 (gene *Ptprc*) positivity and thus we were unable to remove the coexpressed transcriptomes of ribosomal protein transcriptomes for lymphoid cells, which if removed, would have enabled correct classification of myeloid and lymphoid cells among major brain cells in terms of intensity and distribution. Nevertheless, visual/manual curation by surveying individual transcriptomes helped to remove absurdly intense and unrealistically distributed transcriptomic signatures. When we used only the data of Schafflick et al. [68]. , we could not correct the inappropriate signature for B and T-cell compartments even after NSForest application to their data. The data came to look realistic after we adopted cross-tissue data and visual curation upon the two reports by Eraslan et al. [69] and Dominguez Conde et al. [71]. DEG data with an arbitrary threshold of 2.0 higher or -2.0 lower log fold change (LFC) for MHC + infiltrating immune macrophages (Mϕs) or LYVE + infiltrating integrity Mϕs produced 200 or more or 100 or more transcriptomes, respectively. We needed to remove, upon visual curation, 30% or 20% of transcriptomes to annotate the infiltrating monocyte-derived Mϕ. Infiltrating Mϕ and border-associated Mϕ [67, 68] should have been differentiated but this was not possible due to the lack of clear distinction between the two cell types in the literature and the sparsity of the cells of both types. Tissue-resident and effector memory cells were traced with the transcriptomic signature by Dominguez Conde et al. [71]. As these authors included a variety of tissues (unfortunately, brain was not included) and stromal tissue specificity was considered a possible confounder in common for every tissue and thus, as expected, they yielded the signature for three compartments of T/ILC, B-cell and myeloid compartments. Of course, the types/subtypes of classically well-known immune cells belonging to these three compartments represented well the rare immune cells that would have originated from the bone marrow. We found differences in the intensity and distribution of the three compartments in the brain sections between 5xFAD mice and wild-type mice (Fig. 3). Drug/treatment effects should have been disclosed with this comparison, but we simply state that further investigation is warranted with a larger number of mice to avoid confounding factors which may hide or spuriously render probable false-negative/positive results regarding the effect of any tentative immunomodulatory treatments (Supplementary Tables 5 and 6).

The ultimate objective of using ST brain imaging with its visual and quantitative analysis is to convincingly designate the target cells, either major or rare, with regional localization; this can be for either brain parenchymal/stromal or rare immune cells, either resident or infiltrating immune cells and their homeostatic/reactive states, and target genes with significant contributions to pathologic changes in cells/regional tissues and their response to effective or ineffective treatments. More importantly, we could be sure that the unfound cells and transcriptomes were innocent, meaning that they were not affected by the test trial of a novel immunomodulatory therapy. For neuroimmune interactions during the disease process or in response to disease modifying drugs, we now know that the skull BM communicates with the dural sinus and peri-sinus regions, dural lymphatics as well as across ABC and CSF and thus perivascular spaces and ISF; communication can also take a totally different and unique route via the capillary endothelium and stroma of the choroid plexus, and choroid epithelium despite its tight junctions as well as the brain blood vessels’ microvascular endothelium despite its tight junctions. Once immune cells from the three compartments of T/ILCs, B cells and myeloid cells infiltrate the brain parenchyma, dynamically changing along the aging or disease process (in 5xFAD or P301L mice), they can respond to systemic immunomodulatory drug treatment directly or at least indirectly. The abundance of T cells (average 4/mm3) relative to neurons (90,000/mm3) or microglia (6,500/mm3) suggests that a few immune cells could change the response of major brain cells by significantly changing the transcriptomic signature of major brain cells. How the signals are transferred and/or translated from systemically administered anti-CD4 immunoglobulins or NK cell supplements should be investigated further. In this study, the study scheme and analysis methods were proposed to be applied to use ST brain imaging to investigate the impact of novel tentative disease-modifying treatments on neurodegenerative diseases and to elucidate whether regional brain cell-type/state-specific changes in the entire transcriptome per spot/region/cells of the brain or immune system would respond. The comprehensiveness and resolution of the results will be much improved with more novel technology [54, 55] that will be available soon in many institutions, such as Visium methods [47]. Accordingly, methodology for analyzing spatial transcriptomics can be incorporated into high-resolution ST technologies to determine changes in cell types and abundance of rare immune cells with greater confidence.

## Materials and methods

### AD models at different ages

Three-month- and 7.5-month-old male 5xFAD mice (Tg6799; on a C57BL/6-SJL background) containing five FAD mutations in human APP (the Swedish mutation, K670N/M671L; the Florida mutation, I716V; and the London mutation, V717I; and the PS1 mutations M146L/L286V) and wild-type mice were used for spatial transcriptomic brain imaging data. Six- and seventeen-month-old male tau P301L mice (MAPT P301L mutations; on an FVB/N background) were used. Mice of all strains were raised in a laboratory cage with controlled temperature and humidity and on a 12 h light-dark cycle with free access to food and water. All experimental protocols and animal usage were approved (SNU-181018-6, SNU-190221-1-5) by the Institutional Animal Care and Use Committee (IACUC) at Seoul National University. All animals were handled in accordance with the Animal Research: Reporting of in vivo Experiments (ARRIVE) guidelines (https://arriveguidelines.org). Details are in Supplementary Notes.

### Peripheral CD4 T-cell blockade in the 5xFAD AD model

Anti-CD4 antibody (0.5 mg/mouse; Bio X Cell) was intravenously injected into 6.5-month-old 5xFAD and wild-type mice according to group. Samples of different tissues were obtained after a month. Coronal sections of brain samples were used for spatial transcriptomic data acquisition.

### Administration of NK cell supplement in the 5xFAD AD model

NK cells were expanded for 7 days after the isolation of NK cells from BALB/c mouse spleens. NK cells (2 × 10^6^ cells/mouse in saline) were intravenously administered once a week for a total of five times to 6.5-month-old 5xFAD and wild-type mice. Brain samples were obtained after five weeks and used for spatial transcriptomic data.

### Spatial gene expression library construction

Mice were anesthetized with isoflurane inhalation and perfused intracardially with cold DPBS (Dulbecco’s Phosphate-Buffered Saline; Gibco). Then, whole brains were removed. Brain hemispheres were prepared in frozen blocks using OCT compound (Sakura) and cryosectioned into 10 μm coronal and sagittal sections. According to the manufacturer’s protocols using Visium Spatial Tissue Optimization slides (10X Genomics), the permeabilization time was optimized to 12 min. The brain sections were methanol-fixed, hematoxylin and eosin (H&E)-stained and imaged on a TissueFAXS PLUS (TissueGenostics). The slides were merged into a picture of the whole brain using TissueFAXS imaging software. Then, the sections were permeabilized and processed to obtain cDNA Visium Spatial Gene Expression libraries according to the manufacturer’s protocol. To verify the size of PCR-enriched fragments, the template size distribution was checked using a high-sensitivity DNA assay (Agilent Technologies 2100 Bioanalyzer).

### Generation of the count matrix

The libraries were sequenced using HiSeqXten (Illumina) with a read length of 28 bp for read 1 (Spatial Barcode and UMI), 10 bp index read (i7 index), 10 bp index read (i5 index), and 90 bp for read 2 (RNA read). Raw FASTQ data and H&E images were processed by the Space Ranger v1.1.0 (10X Genomics) pipeline for the gene expression analysis of Visium Spatial Gene Expression library data. Illumina base call files from the Illumina sequencing instrument were converted to FASTQ format for each sample using the ‘mkfastq’ command. Visium spatial expression libraries were analyzed with the ‘count’ command. Image alignment to predefined spots was performed using the fiducial alignment grid of the tissue image to determine the orientation and position of the input image. Sequencing reads were aligned to the mm10 reference genome (mm10-2020-A) using STAR (v2.5.1b) aligner. Gene expression profiling in each spot was performed with unique molecular identifier (UMI) and 10X barcode information.

### Integration and spot clustering

A total of 63 spatial transcriptomic datasets, including brain tissue from wild-type and 5xFAD mice, with 32,885 genes in common were integrated and analyzed. The generated gene counts were normalized using ‘LogNormalize’ methods with a scale factor of 10,000. The top highly variable genes (HVGs; *n* = 2,000) in each tissue slide were identified using the variance stabilizing transformation (vst) method. The 2000 integration genes across all slides were then selected by ranking the genes by the number of slides in which they are variable in and their median rank of variability across the slides. For each slide, the log-normalized count matrix for the selected genes was scaled and the total RNA counts in each spot was regressed to remove the influence of the total count in the integration process. Principal component analysis (PCA) was performed for dimensionality reduction. Integration was performed for multiple spatial datasets prior to spot clustering to remove the batch effect. To flexibly integrate a large number of slides with both coronal and sagittal sections, reciprocal PCA (RPCA) was used to discover a set of anchors between the datasets, and normal (wild-type) mice were used as a reference during integration. The anchors were utilized to correct the count matrix in each spatial spot. The corrected counts were then scaled and PCA was performed. For spot clustering, a shared nearest neighbor (SNN) graph was constructed and graph-based clustering was performed using the Louvain algorithm. The resulting spot clusters were visualized using two different approaches: spatially mapped to the tissue based on spatial barcodes, or plotted in 2-dimensional space using Uniform Manifold Approximation and Projection (UMAP). The optimal resolution of the spot clusters was determined by decreasing the resolution value and visually examining the appropriate granularity of the spatial clusters that corresponded well to the anatomical structure. The anatomical location of each cluster was visually determined by comparison with the Allen Mouse Brain Reference Atlas (https://mouse.brain-map.org/static/atlas). As a result, the resolution was set to 0.15 for subsequent analysis. All analyses were performed using the R package, Seurat (version 4.1.1) [74].

### Differential gene expression analysis

MAST (Model-based Analysis of Single-cell Transcriptomics) was used to perform differential gene expression analysis [96]. MAST accounts for the bimodal distribution of counts in the spatial transcriptomics and uses a generalized linear model with the proportion of genes expressed in each spot as a covariate to model the normalized counts. Differentially expressed genes (DEGs) were extracted from the comparison of wild-type and 5xFAD mice in each spot cluster defining the anatomical region in the brain. The cutoff for significantly different genes was false discovery rate (FDR)-adjusted *p* < 0.05 and log FC > 0.25.

### Overrepresentation analysis

Overrepresentation analysis was performed and the Gene Ontology (GO) biological process terms associated with DEGs were identified. The count ratio was defined as the ratio of the proportion of the genes constituting GO terms among the DEGs to the proportion of genes constituting GO terms among total genes. Statistical significance was calculated based on the hypergeometric model, and correction for multiple comparisons was performed using the Benjamini-Hochberg procedure. The dot plots for the significant GO terms were drawn by showing the number of overlapping genes between the DEGs and each GO term, the count ratio, and the adjusted p-values. Overrepresentation tests were performed using clusterProfiler [118], which supports statistical analysis and visualization of functional profiles for gene sets. The packages ‘enrichplot’ and ‘igraph’ were additionally used to visualize the results.

### Marker panel selection and curation

To analyze the spatial patterns of major cell types and immune cell types, the panel of marker genes was constructed and curated for each cell type. For the cell types identified in studies not using scRNAseq, individual genes were determined based on reference papers. For the cell types defined by scRNAseq, Necessary and Sufficient Forest (NSForest) version 2 [59] was applied and signature genes for the cell type were determined based on the cell type annotation information. The NSForest algorithm scores genes according to binary expression profiles in a specific cell type compared to other cell types. Then, based on the random forest algorithm, the minimum gene set that best describes the given cell type was searched. After selecting signature genes based on NSForest, the gene sets were refined to exclude the genes that are highly expressed in major cell types. This is particularly important when the scRNAseq data represent a subpopulation of the cells in the brain, such as immune cell sorted datasets. As a validation process, the spatial expression of the selected marker genes was examined and the genes were excluded if they showed a non-specific distribution pattern for the cell type. As a final step, genes that were not present in our spatial transcriptomic data were excluded. The curated gene sets are listed in Supplementary Table 7.

### Comparison of cellular signatures across groups

After curation of the marker panel, a gene set that best represents a particular cell type, the signature score of each cell type was computed on the spatial transcriptomic data by utilizing the AddModuleScore function in Seurat [44] with default parameters. For each gene in the gene set, a fixed number of control genes with the same average expression level as the gene were randomly selected. The difference between the average expression of the gene set and that of the control gene sets was calculated and named the cell signature score. The score in each spot was spatially mapped to the tissue using the SpatialFeaturePlot function in Seurat, and the spatial distribution pattern was identified. The average of the signature scores in a given region of interest was calculated and the values were compared between groups using the Wilcoxon rank-sum test. Correction for multiple comparison was performed using the Bonferroni method. The cutoff for the adjusted p-value was 0.05.

### Cell type deconvolution analysis

The cell type distribution represented by the cell signature scores was compared to that derived by the cell type deconvolution method, CellDART [46]. CellDART first trains a model to extract cell type proportions from the synthetic mixture of cells generated from the reference scRNAseq dataset, and then adapts the model to predict the cell type composition of the spot, which is a mixture of multiple cells. For the major brain cell types, snRNAseq datasets from mouse brain coronal slices [49] were used as a reference for predicting spatial cell distribution. However, in the case of immune cells, the majority of scRNAseq datasets are obtained after cell sorting strategies such as fluorescence-activated cell sorting (FACS), and there is a mismatch in cell type and composition between spatial and single-cell datasets. Therefore, the cell type deconvolution tool spSeudoMap was used to compensate for this discrepancy [50]. For lymphoid and myeloid brain cell types, scRNAseq samples from CNS border immune cells were used [119], and for microglia, scRNAseq samples from brain immune cells were used [89]. The distribution of representative cell types that showed significant differences between wild-type and 5xFAD mice was evaluated: homeostatic microglia, reactive microglia, macrophages, monocytes, dendritic cells, innate lymphoid cells, natural killer cells, and T cells. The cell type annotation information from the reference single-cell dataset was used for the deconvolution analysis. Default parameter values suggested in the user manual were applied for the analysis.

### Statistical analysis

For the spatial transcriptomic data, plots in R were created either with the ggplot2 R package or Seurat modified by custom codes for data visualization. All p-values reported in this study were adjusted by FDR (for DE analysis using MAST) using Benjamini-Hochberg procedure or Bonferroni method (all other analyses). The p-values below 0.05 were considered statistically significant.

### Development of an application to visualize and quantify ST datasets

An R shiny-based application named STquantool was developed to comprehensively analyze ST datasets to explore cell type- and cell state-specific regional changes in wild-type, 5xFAD, and treatment mouse models. The application allows users to easily load and integrate the multiple ST datasets and visualize the spatial expression of genes and cell type scores based on Seurat [74] and shiny running on R (ver. 4.1.1). One of the key features of STquantool is that it facilitates the curation of cell type-specific marker combinations by sorting out key genes based on the NSForest [59] algorithm and finalizing the markers by visually assessing the spatial expression patterns. As an adjunct, the cell type decomposition method CellDART [46] can be implemented to find the spatial distribution patterns of major cell types constituting brain tissues. Moreover, the spatial patterns of the cell scores and cell fraction can be quantified and statistically analyzed with STquantool. Finally, the gene-level transcriptomic alterations between the mouse groups can be explored by performing the DEG analysis provided in the application. Then, the functional implications of the selected genes can be represented by gene ontology (GO) and Kyoto Encyclopedia of Genes and Genomes (KEGG) terms [120–122]. The suggested platform was packaged and can be readily installed from GitHub (https://github.com/bsungwoo/STquantool.git).

### Electronic supplementary material

Below is the link to the electronic supplementary material.

Supplementary Material 1

Supplementary Material 2

Supplementary Material 3

## Data Availability

All data are available in the main text or the supplementary materials. Additionally, the Visium spatial transcriptomics datasets utilized in this research are accessible on the data repository (https://zenodo.org/records/10404412). The spatial transcriptomics analytical pipeline, STquantool, is available for installation from GitHub at https://github.com/bsungwoo/STquantool.git
