# Supplementary material for: Spatial transcriptomic brain imaging reveals the effects of immunomodulation therapy on specific regional brain cells in a mouse dementia model
Source: BMC Genomics. 2024 May 25;25:516. doi: 10.1186/s12864-024-10434-8 (PMC11128132; doi:10.1186/s12864-024-10434-8)
Supplement: Supplementary file 3 — Supplementary Material 3 [file 12864_2024_10434_MOESM3_ESM.docx]

**Supplementary Notes**

We reviewed our procedure in methods and added the comments according to the ARRIVE Essential 10 ([https://arriveguidelines.org](https://arriveguidelines.org/)).

**Study design**

In this investigation, we compared 7-month-old wild type and 5xFAD mice to identify the changes in spatial transcriptomic signatures and brain cell types using the 10x Genomics Visium platform. As the wild type mice, C57BL/6-SJL mice, which had no mutant genes as a result of genotyping analysis, were used. For 5xFAD mice, the same background mice containing mutated genes were used.

**Sample size**

For ethical reasons, we tried to reduce the number of mice as small as possible, but for acquisition of the sufficient number to discover the true difference between groups, we needed to increase the number of mice as large as affordable. Detailed numbers of animals used are charted in Supplementary Figure 1. Sample size was determined considering statistical significance between two groups using Benjamini-Hochberg or Bonferroni methods.

**Inclusion and exclusion criteria**

The 5xFAD mice were included in the study for the Visium analysis study if the Y-maze behavioral assay showed an alternation ratio of less than 50%. The mice were excluded for the behavior analysis if the mice had no mutant genes as a result of genotyping analysis, or if the mice died prematurely.

**Randomization**

For Y-maze behavior analysis, mice identified as 5xFAD mice through genotyping were randomly selected, and mice for Visium analysis were also randomly selected among mice showing 50% alternation rate as a result of the Y-maze.

**Blinding**

During Y-maze behavior analysis, we performed the maze test blindly by masking groups of mice. However, investigators could not be blinded while collecting brain samples and analyzing the dataset for Visium between wild type and 5xFAD mouse groups. Instead, all materials and machinery to obtain the Visium dataset were used identically for both groups, and the same statistical measures were applied.

**Outcome measures**

Alternation scores for Y-maze were measured as establish standard. Especially, acclimation the day before and the measurement ensured the reproducibility and thus the variability of alternation scores per individual mice represent the biological variability of individual mice.

**Statistical measures**

For the spatial transcriptomic data, plots in R were created either with the ggplot2 R package or Seurat modified by custom codes for data visualization. All p-values reported in this study were adjusted by FDR (for DE analysis using MAST) using Benjamini-Hochberg procedure or Bonferroni method (all other analyses). The p-values below 0.05 were considered statistically significant.

**Experimental animals**

Three-month- and 7.5-month-old male 5xFAD mice (Tg6799; on a C57BL/6-SJL background) containing five FAD mutations in human APP (the Swedish mutation, K670N/M671L; the Florida mutation, I716V; and the London mutation, V717I; and the PS1 mutations M146L/L286V) and wild-type mice were used for spatial transcriptomic brain imaging data. Further investigation is open in female and mice of different ages.

**Experimental procedures**

Technical establishment was reported in the main text, especially for the behavior analysis, drug administration, Visium data generation, and spatial sequencing data analysis.

**Results**

Details of results are referred to the main text, To avoid redundancy and to maintain succinctness of description we would not comment any further here in this supplementary note.
